# Antigen-Specific CD4^+^CD8^+^ Double-Positive T Cells Are Increased in the Blood and Spleen During *Ehrlichia chaffeensis* Infection in the Canine Host

**DOI:** 10.3389/fimmu.2018.01585

**Published:** 2018-07-11

**Authors:** Jodi L. McGill, Ying Wang, Chanran K. Ganta, Gunavanthi D. Y. Boorgula, Roman R. Ganta

**Affiliations:** ^1^Department of Veterinary Microbiology and Preventative Medicine, College of Veterinary Medicine, Iowa State University, Ames, IA, United States; ^2^Center of Excellence for Vector-Borne Diseases, Department of Diagnostic Medicine/Pathobiology, College of Veterinary Medicine, Kansas State University, Manhattan, KS, United States

**Keywords:** *Ehrlichia chaffeensis*, *Rickettsia*, vaccination, immune response, double-positive T cells, tick-borne disease, adaptive immune response

## Abstract

*Ehrlichia chaffeensis* is an obligate intracellular bacterium belonging to the order, Rickettsiales and is a frequent cause of severe and fatal tick-borne infection in people in North America. The reservoir host for *E. chaffeensis* is the white-tailed deer, while humans and dogs are regarded as common incidental hosts. In dogs, we and others have shown that *E. chaffeensis* establishes a chronic infection that persists for several weeks to months, while promoting the development of Th1 and Th17 cellular responses and pathogen-specific humoral immunity. We demonstrate here that vaccination with a live, attenuated clone of *E. chaffeensis* bearing a targeted mutation in the Ech_0230 gene neither promotes the development of long-lived cellular or humoral immunity, nor confers protection against secondary wild-type *E. chaffeensis* challenge. In dogs, a population of mature CD4^+^CD8^+^ double-positive (DP) T cells exists in the periphery that shares similarities with the DP T cell populations that have been described in humans and swine. Little is known about the function of these cells, particularly in the context of infectious diseases. Here, we demonstrate that canine DP T cells expand significantly in response to *E. chaffeensis* infection. Using *in vitro* antigen recall assays, we further demonstrate that canine DP T cells undergo clonal expansion, produce IFNγ and IL-17, and upregulate expression of granzyme B and granulysin. Together, our results demonstrate that DP T cells accumulate in the host during *E. chaffeensis* infection, and suggest that alternative lymphocyte populations may participate in the immune response to tick-borne infections in the incidental host.

## Introduction

*Ehrlichia chaffeensis* is a Gram-negative, obligate intracellular bacterium. It is a member of the order Rickettsiales, in the family Anaplasmataceae. It is the causative agent of human monocytic ehrlichiosis (HME) (1–3). HME causes significant morbidity, with 40–60% of reported cases requiring hospitalization, and mortality in 3–5% of infected individuals (4, 5). Poor outcomes due to HME are frequently attributed to delays in diagnosis and treatment, as well as infection in children and immunocompromised individuals (6). *E. chaffeensis* is an obligate intracellular pathogen that is primarily transmitted by the lone star tick, *Amblyomma americanum* (2). White-tailed deer are regarded as the reservoir hosts for *E. chaffeensis*, while humans, dogs, and other vertebrate species are considered incidental hosts. Currently, treatment options for *E. chaffeensis* infection are limited to a single class of tetracycline antibiotics, and there is no approved vaccine for use in humans or animals.

Vaccine development, and our knowledge of disease pathogenesis and immunity, has been severely limited by the lack of suitable animal models for *E. chaffeensis* infection. Mice in the wild do not appear to contract *E. chaffeensis* (3); and the pathogen is poorly infectious in experimental challenge settings in this host. Therefore, our laboratory uses a model of *E. chaffeensis* infection in dogs (7–11). Dogs infected with *E. chaffeensis* develop ehrlichemia that is detectable within 3 days after infection and the infection persists for several weeks to months (7–12). Dogs display clinical symptoms, with fever and thrombocytopenia (7, 9, 11, 12); and develop similar disease pathology as reported in humans and in the reservoir host, white-tailed deer (2, 11, 12). Similar to humans, dogs are also an outbred species that is naturally susceptible to *E. chaffeensis* infection. Thus, our experimental *E. chaffeensis* infection studies in dogs provide an ideal opportunity to study disease pathogenesis and immunity, and to develop novel vaccines and therapeutics.

We have recently reported methods for the generation of both random and targeted mutations in *E. chaffeensis*; and through our mutagenesis efforts, we generated a panel of mutant organisms that display defects in their capacity to replicate in a vertebrate host (13). One of these mutant clones, containing a transposon insertion in the Ech_0660 gene, was found to induce a strong immune response in white-tailed deer and dogs, and we recently reported that vaccination with the live, attenuated Ech_0660 mutant organisms conferred protection from both needle- and tick-transmitted wild-type *E. chaffeensis* infection in dogs (9, 10). In addition to the Ech_0660 mutant clone, we also generated a mutant organisms containing a transposon mutation in the gene encoding for Ech_0230, which displayed similar defects in its capacity to replicate *in vivo* in the vertebrate host (13). Given our previous success with the live, attenuated Ech_0660 mutant, we hypothesized that exposure to the attenuated Ech_0230 mutant would induce *E. chaffeensis-*specific immunity in the dog, and thus confer protection from virulent infection. We have recently reported improved methods for performing targeted mutagenesis experiments in *E. chaffeensis* (14). Therefore, using our targeted mutagenesis strategy, we generated a mutant strain of *E. chaffeensis* with an Ech_0230 gene inactivation, and determined if vaccination with the Ech_0230 mutant confers protection from secondary infection challenge with wild-type *E. chaffeensis*.

Previous reports in the murine model have demonstrated that immunity to *E. chaffeensis* infection can be mediated by both antibody and cellular immune responses (15–22). T helper 1 (Th1) type immunity is likely one of the most important responses for control and clearance of a primary *E. chaffeensis* infection as judged from the studies carried out in the murine host (16, 19, 20). Using the canine host model, we recently demonstrated that *E. chaffeensis-*infected animals also mount a strong Th17 response (10). In addition to the role of classical CD4 T cells, however, there are a number of other immune populations that can contribute to resistance or pathogenesis during *E. chaffeensis*, including NK cells (23), NKT cells (24, 25), and CD4^−^CD8^−^ double-negative T cells (20). Given that we know little about the immune response to HME in a natural host, we are particularly interested in further defining the immune components that may play a role in disease resistance and susceptibility.

CD4^+^CD8^+^ double-positive (DP) T cells have been described in a number of species, including mice (26), pigs (27), monkeys (28), and humans (29). In swine, DP T cells are a well characterized, polyfunctional, memory population that is thought to contribute to resistance to viral infections (30). CD4^+^CD8^+^ DP T cells are often expanded in human patients with chronic diseases, such as HIV infection (31) and certain cancers (32, 33). In dogs, there have been only a few reports describing DP T cells (34–38), and little is known about the role of this population in the immune response to infectious diseases. Here, we describe for the first time, significant expansion of a CD4^+^CD8^+^ DP T cell population in dogs infected with *E. chaffeensis*, and report the capacity for this population to undergo clonal expansion, secrete IFNγ and IL-17, and upregulate expression of the cytotoxicity-associated molecules granulysin and granzyme B in specific response to *E. chaffeensis* antigen.

## Materials and Methods

### Creation of Ech_0230 Gene Disruption Mutant by Homologous Recombination

A targeted disruption mutation was created in the Ech_0230 gene of *E. chaffeensis* Arkansas strain. The mutant was generated by allelic exchange using a linear construct fragment consisting of 1 kb genomic regions as homology arms at each end flanking *Tuf*-aadA antibiotic cassette and the clonal purity of the mutant was verified (14).

### *In Vitro* Cultivation of *E. chaffeensis*

The Ech_0230 targeted mutant organisms for the vaccination experiment were continuously cultivated in the ISE6 tick cell line, an *Ixodes scapularis* embryonic cell line; the wild-type *E. chaffeensis* Arkansas isolate for the challenge experiment was cultured in the canine macrophage cell line (DH82) as described previously (39).

### Animal Infections

Ten male, purebred beagle dogs of 6–8 months of age were purchased from Covance Research Products (Denver, PA, USA). Male dogs were used in these studies because of their higher body weight, which allows for increased blood volume collection without endangering the health of the animal. In prior studies we have used mixed genders and have observed no differences in the immune response or the course of disease between male and female dogs (7–11, 13, 40). Animals were housed in a climate-controlled, biosafety level-2 facility at Kansas State University. All dogs were allowed to acclimate for 5 days prior to vaccination. Experimental procedures were performed in strict compliance with federal and institutional guidelines and were approved by the Kansas State University Institutional Animal Care and Use Committee.

Intravenous vaccination with attenuated *E. chaffeensis* homologous recombination mutant Ech_0230 in dogs was performed as previously described (7, 9). Briefly, Ech_0230 mutant *E. chaffeensis* cultures were collected at ~80–90% infectivity. Animals (group 1; *n* = 5) were inoculated i.v. with ~2 × 10^8^ mutant organisms suspended in 1 ml phosphate buffered saline (PBS). Control groups remained unvaccinated. On day 28 post-vaccination, the group 1 animals were challenged by intravenous inoculation with ~2 × 10^8^ wild-type *E. chaffeensis* organisms grown in DH82 cells. Three dogs that had not previously received Ech_0230 were similarly challenged with wild-type *E. chaffeensis* organisms to serve as the unvaccinated infection controls (group 2), and two dogs were maintained as uninfected controls (group 3). Group 3 animals were housed independently of the infected dogs and were handled before the animals in groups 1 and 2. All animals were humanely euthanized by barbiturate overdose on day 28 post-challenge and a board-certified veterinary pathologist conducted a complete necropsy on all animals. The gross necropsy and histopathology analysis, including the grading system, was performed as described in our previously published study (11).

### Evaluation of Dog Blood for Infection by PCR

About 2 ml each of peripheral blood was collected *via* the cephalic vein in sterile EDTA tubes on day zero (prior to infection) and on days 3, 7, 10, 14, 17, 21, 24, and 28 post-vaccination. Similarly, blood samples were collected on days 3, 7, 10, 15, 17, 21, 24, and 28 post-challenge.

The blood samples were stored at 4°C until use (up to a maximum time of 3 days). Blood samples were spun at 3,000 rpm in a Clay Adams Sero-fuge (Becton Dickinson, Sparks, MD, USA) for 5 min. Plasma was removed and about 1 ml of buffy coat each was transferred to a 15 ml sterile Falcon centrifuge tube containing 10 ml RBC lysis buffer (155 mM NH_4_Cl, 10 mM KHCO_3_, and 0.1 mM EDTA) and mixed several times until complete lysis of erythrocytes. The samples were then centrifuged at 5,000 *g* for 5 min and the supernatants were discarded. The buffy coat pellet from each sample was resuspended in 300 µl of 1 × PBS. One hundred µl each of the buffy coats from dog blood were used for isolating total genomic DNA by using the Wizard SV Genomic DNA purification kit as per the manufacturer’s instructions (Promega, Madison, WI, USA); purified DNA from each sample was stored in 100 µl of buffer containing 10 mM Tris–HCl and 1 mM EDTA (pH 8.0) (TE buffer).

The DNA samples were used to assess *E. chaffeensis* infection status by performing semi-nested PCR targeting the Ech_1136 gene encoding for the p28-Omp 14 protein or the insertion-specific region of the mutant Ech_0230 clone as previously described (9). Primers used in insertion-specific PCR are: RRG1944 (forward): 5′-ATTAGTGCTATGGCATTTGGTC, RRG1596 (reverse): 5′-AAACAAATACCTTTAACATCATTAAACCATTTC, and RRG1254 (forward nested): 5′-GTGGATTGCTTATAGGAGCAATAGG. Briefly, 2 µl of genomic DNA from the dog blood was used for the first round of PCR reactions in a 25-µl reaction volume using Platinum Taq DNA polymerase per the manufacturer’s instructions (Life Technologies, Grand Island, NY, USA). The PCR reactions were performed in a GenAmp9700 instrument (Applied Biosystems, Foster City, CA, USA) with the following temperature cycles: 94°C for 4 min; 35 cycles of 94°C for 30 s, 52°C for 30 s, and 72°C for 1 min; and 1 cycle of 72°C for 3 min. The second round of PCR reactions was performed using the same cycling conditions as those for the first-round PCR, and the templates for the second round included 2 µl of 1:100 diluted products from the first PCR with a nested PCR primer set. The PCR products were resolved on 1.5% agarose gel to identify specifically sized products.

### Enzyme-Linked Immunosorbent Assay (ELISA) for *E. chaffeensis-*Specific IgG

Plasma samples collected prior and following infection were assessed by ELISA for the presence of *E. chaffeensis*-specific IgG as previously reported (7). Briefly, 96-well plate was coated with 20 ng/well of host cell-free *E. chaffeensis* whole-cell lysates or cell lysates from uninfected DH82 cells (negative control wells). Plasma samples were diluted 1:50 and used as the primary antibody. HRP-conjugated goat anti-dog IgG (1:50,000) was used as a secondary antibody. The absorbance was measured at 450 nm by ELISA plate reader. All assays were performed in duplicate wells and are presented as the mean value.

### Preparation and Culture of Peripheral Blood Mononuclear Cells (PBMC) and Splenocytes

Peripheral blood mononuclear cells were prepared as previously described (10). Briefly, cells were isolated by density centrifugation from buffy coat fractions of peripheral blood collected into 2× acid citrate dextrose. Cells were washed and resuspended in complete RPMI (cRPMI) composed of RPMI-1640 (Gibco, Carlsbad, CA, USA) supplemented with 2 mM l-glutamine, 25 mM HEPES buffer, 1% antibiotic–antimycotic solution, 50 mg/ml gentamicin sulfate, 1% nonessential amino acids, 2% essential amino acids, 1% sodium pyruvate, 50 µM 2-mercaptoethanol, and 10% (vol/vol) fetal bovine serum. Splenocytes were prepared by pressing approximately 1 g of spleen tissue through a tissue sieve to prepare a single-cell suspension. Cells were then washed in cRPMI, enumerated, and surface stained for flow cytometry as described below.

For lymphocyte proliferation assays, PBMCs were labeled with 1 μM CellTrace Violet (Life Technologies Inc.) per manufacturer’s instructions. Cells were cultured for 5 days at 37°C with 4 × 10^5^ cells/well in 96-well plate and were stimulated with 10 µg/ml host cell-free *E. chaffeensis* whole-cell lysate that was grown in ISE6 tick cells. As a positive control, cells were stimulated with 5 µg/ml Concanavalin A (Sigma-Aldrich) for 5 days at 37°C. Mock-stimulated control cultures were included to correct background proliferation or cytokine production. Background proliferation levels in mock-stimulated cultures were approximately 5–10%.

### Antibodies and Flow Cytometry

The following monoclonal antibodies were used in these studies: mouse anti-canine CD3-FITC or APC-Cy7 (clone CA17.2A12), mouse anti-canine CD4-RPE-Cy7 (clone YKIX302.9), mouse anti-canine CD8-Alexa Fluor 647 (YCATE55.9), purified mouse anti-canine CD25 (clone P4A10), and mouse-anti-bovine IFNγ-RPE (clone CC302) all from AbD Serotec (Raleigh, NC, USA); mouse anti-canine CD44 (clone H22A) and mouse-anti-bovine CD14 (clone CAM36A) from Washington State University; mouse anti-human CD49d-RPE (clone 9F10) and mouse anti-human CD11a-Alexa Fluor 488 (clone HI111) from Biolegend; and mouse anti-human IL-17A-APC (clone eBio64DEC17) from eBioscience. The following secondary antibodies were used: goat anti-mouse IgG2a-PerCPCy5.5 from Biolegend and goat anti-mouse IgG1-APC-Cy7 from Southern Biotech.

For surface staining, cells were resuspended at 10^7^ cells/mL in FACS buffer (0.1% NaN_3_, 10% fetal calf serum, and PBS) and incubated for 20 min at 4°C with 10 µg/ml primary antibodies or as recommended by the manufacturer. If required, cells were resuspended in FACS buffer with 5 µg/ml secondary antibodies and incubated for 20 min at 4°C. Cells were washed and fixed in BD FACS Lysis buffer (BD Biosciences) per manufacturer’s recommendation.

Intracellular cytokine staining for IFNγ and IL-17 was carried out using the BD Fixation and Permeabilization Solution kit with GolgiPlug (BD Biosciences). Cells were cultured with antigen overnight with GolgiPlug (Brefeldin A). Cells were surface stained as above, then fixed, permeabilized, and stained for intracellular IFNγ (Clone CC302, 10 µg/ml) or IL-17 (Clone eBio64DEC17) per manufacturer’s instructions.

Flow cytometry data were collected on a BD LSR Fortessa X-20 flow cytometer and analyzed using FlowJo software (Tree Star Inc., San Carlos, CA, USA). For analysis, gating was determined using isotype control antibodies and fluorescence minus one controls. Positive results for intracellular cytokine staining and cell proliferation were corrected for background activation by subtracting the frequency of cells that divided or expressed cytokine in mock-stimulated cultures. Typical levels of background proliferation were ~5–10%, while background levels of cytokine production were ~1–5%.

### Purifying DP T Cells

For sorting, cells were resuspended in cRPMI and then surface stained with CD3, CD4, and CD8 as described above. Cells were then sorted to >95% purity based upon expression of all three markers, using a BioRad S3 Cell Sorter. Autologous CD14^+^ monocytes were isolated from the peripheral blood using magnetic activated cell separation, using a protocol we have previously published for bovine monocytes (41, 42). After sorting, cells were incubated at 37°C with 10^5^ DP T cells/well in 96-well plate, with a ratio of 1 autologous monocyte:5 DP T cells per well, and were stimulated with 10 µg/ml host cell-free *E. chaffeensis* whole-cell lysate that was grown in ISE6 tick cells. As a positive control, cells were stimulated with 5 µg/ml Concanavalin A (Sigma-Aldrich). Mock-stimulated control cultures were included to correct for background proliferation or cytokine production.

### ELISA for Canine Cytokines

Peripheral blood mononuclear cell or DP T cell culture supernatants were collected after 5 days of stimulation with 10 µg cell lysate prepared from host cell-free *E. chaffeensis* grown in the ISE6 tick cell line. IFNγ and IL-17A protein concentrations in the culture supernatants were determined by canine-specific commercial ELISA kits (Canine IFNγ Duoset ELISA and canine IL-17A Duoset ELISA, R&D Systems, Minneapolis, MN, USA) per manufacturer’s instructions.

### Real-Time PCR

Total RNA was extracted using the RNeasy Mini RNA Isolation kit (Qiagen) according to manufacturer’s instructions. Contaminating genomic DNA was removed using the RNase-Free DNAse Digestion set (Qiagen). Total eluted RNA was reverse transcribed into cDNA using Superscript III Reverse Transcriptase and Random Primers (both from Life Technologies, Inc.). Real-time PCR was performed using Power SYBR Green PCR Master Mix (Applied Biosystems). The primer sequences for canine GAPDH, canine granzyme B, and canine perforin have been previously published (43, 44). Forward and reverse primers were designed for canine granulysin (Accession XM_845424.3) using PrimerQuest software from Integrated DNA Technologies: canine granulysin (forward): 5′-TGTGTAGTGTTGCCCAGTTT-3′; canine granulysin (reverse): 5′-CTCCTTGGACACCTACTTGATG-3′. The reactions were performed on an Agilent MX3000P Real-Time PCR machine (Agilent) with the following cycling conditions: 2 min at 50°, 10 min at 95°, followed by 40 cycles of 15 s at 95° and 1 min at 60°, and a dissociation (melting) curve (15 s at 95°, 1 min at 60°). Relative gene expression was determined using the 2^−ΔΔCt^ method, with GAPDH as the reference housekeeping gene.

### Statistics

Statistical analysis was performed using Prism v6.0f software (Graphpad Software, Inc.). To account for time and repeated measures, antibody and T cell responses were analyzed using a two-way ANOVA with Sidak’s multiple comparisons test. DP T cell results (proliferation and cytokine production) were analyzed using a one-way ANOVA with Tukey’s multiple comparisons test. Experiments using sorted DP T cells were analyzed using student’s *t-*test. For proliferation and intracellular cytokine staining data, background (mock) responses were subtracted from the response to antigen and results are presented as change over mock.

## Results

### A Disruption Mutation in the Ech_0230 Gene Causes Attenuation in Animals

In our previous study, we reported a disruption mutation in Ech_0230 gene of *E. chaffeensis* by transposon mutagenesis method (13). In a follow-up study, we recently generated a similar mutation by employing a targeted mutagenesis method (14). Transcriptional analysis showed that the disruption mutation caused transcriptional inactivation of the targeted Ech_0230 gene (13, 14). Our prior studies also demonstrated that the Ech_0230 mutation caused attenuation and rapid clearance of *E. chaffeensis* from white-tailed deer and dogs, but not the tick host (8, 13). In the current study, we investigated whether targeted disruption mutation in Ech_0230 similarly could affect the pathogen’s growth and persistence in the incidental host, dog. Five dogs were infected i.v. with 2 × 10^8^ Ech_0230 targeted mutant organisms. Peripheral blood was monitored every 3–4 days post-infection for ehrlichemia by PCR analysis for two target genes, one specific for the Ech_0230 gene disruption insertion, and one specific for the Ech_1136 gene. All animals were negative for ehrlichemia at all time points (Table 1), indicating that the targeted mutant organism was rapidly cleared, and suggesting that the Ech_0230 gene may be required to establish a productive *E. chaffeensis* infection in the mammalian host. This result corroborates our previous studies, where we observed rapid clearance of the organism from white-tailed deer and dogs when using the gene inactivation mutant of Ech_0230 caused by random mutagenesis (8).

**Table 1:** Infection status of dogs vaccinated with the Ech_0230 mutant.

	Dog ID	Target	Days post-vaccination								
			0	3	7	10	14	17	21	24	28
Ech_0230Vaccinated animals (*n* = 5)	Fed 13–16	Ech_0230	−	−	−	−	−	−	−	−	−
		Ech_1136	−	−	−	−	−	−	−	−	−
	Fed 14–16	Ech_0230	−	−	−	−	−	−	−	−	−
		Ech_1136	−	−	−	−	−	−	−	−	−
	Fed 15–16	Ech_0230	−	−	−	−	−	−	−	−	−
		Ech_1136	−	−	−	−	−	−	−	−	−
	Fed 16–16	Ech_0230	−	−	−	−	−	−	−	−	−
		Ech_1136	−	−	−	−	−	−	−	−	−
	Fed 17–16	Ech_0230	−	−	−	−	−	−	−	−	−
		Ech_1136	−	−	−	−	−	−	−	−	−

### Vaccination With the Attenuated Ech_0230 Mutant Does Not Confer Protection Against Wild-Type Infection Challenge in Dogs

We have previously demonstrated that vaccination with the Ech_0660 mutant organism confers protection from both needle- and tick-transmitted wild-type *E. chaffeensis* challenge in the canine host (9, 10). To determine if the attenuated Ech_0230 mutant could similarly confer protection against wild-type infection in dogs, we challenged the Ech_0230 vaccinated dogs (group 1, *n* = 5) with wild-type *E. chaffeensis* on day 28-post vaccination. Three unvaccinated dogs were also challenged with 2 × 10^8^ wild-type *E. chaffeensis* and served as infection controls (group 2). Two control dogs remained uninfected and served as negative controls (group 3). Peripheral blood was collected every 3–4 days following challenge, and analyzed for ehrlichemia by PCR detection analysis targeting to the Ech_1136 gene. We observed no positive PCR results in samples collected from our uninfected control animals (group 3). As seen in Table 2, animals in both groups 1 and 2 were positive for ehrlichemia ~50% of the time, and we observed no significant differences between groups (group 1:19 positives/40 total = 47.5%; group 2, unvaccinated controls: 13 positives/24 total = 54.2%). As in our prior studies, we observed only occasional mild fevers in animals in groups 1 and 2. Similarly, we noted elevated WBC counts on some days post-infection; however, we did not find any significant differences between the Ech_0230 vaccinated animals and the unvaccinated controls. The uninfected control dogs (group 3) did not exhibit any fever or changes to WBC counts. No gross pathologic lesions were noted in any of the infected or control animals. Histopathologic analysis of lungs and spleens from dogs in groups 1 and 2 revealed microscopic lesions that were consistent with that described in our previous study (11), and included small to moderate numbers of perivascular infiltrates of macrophages and lymphocytes in the lungs, and mild to moderate lymphoid hyperplasia in the spleen. No significant microscopic lesions were present in the liver, lymph nodes, heart, muscle, adrenal glands, kidneys, blood vessels, bone marrow, or brain. One animal from group 2 (dog 20) had diffuse interstitial edema in the lungs. The pathologic changes were not significantly different between dogs in groups 1 and 2. One uninfected control dog (group 3) had mild perivascular infiltrates in the lungs, although the cause is unknown, as the animal was consistently negative for *E. chaffeensis* by PCR detection methods. Together, our histopathology results correlate with the results of our PCR analysis, and confirm that Ech_0230 vaccination does not afford significant protection from virulent *E. chaffeensis* infection.

**Table 2:** Infection status of dogs in groups 1–3 following wild-type *Ehrlichia chaffeensis* challenge.

	Dog ID	Target	Days post-challenge							
			0 Day 28	3 Day 31	7 Day 35	10 Day 38	15 Day 43	17 Day 45	21 Day 49	24 Day 52
Group 1 Ech_0230 vaccinated	Fed 13–16	Ech_1136	−	−	−	+	+	−	+	+
	Fed 14–16	Ech_1136	−	+	+	+	−	+	+	+
	Fed 15–16	Ech_1136	−	−	−	−	+	+	−	+
	Fed 16–16	Ech_1136	−	+	−	+	−	−	−	−
	Fed 17–16	Ech_1136	−	−	+	+	+	+	−	−
Group 2 unvaccinated	Fed 20–16	Ech_1136	−	+	−	+	−	+	+	−
	Fed 21–16	Ech_1136	−	−	−	+	+	+	+	−
	Fed 22–16	Ech_1136	−	+	−	+	−	+	+	+
Group 3 uninfected	Fed 18–16	Ech_1136	−	−	−	−	−	−	−	−
	Fed 19–16	Ech_1136	−	−	−	−	−	−	−	−

### Ech_0230 Vaccination Induces Humoral, but Not Cellular Immune Responses in the Canine Host

**Figure 1:**
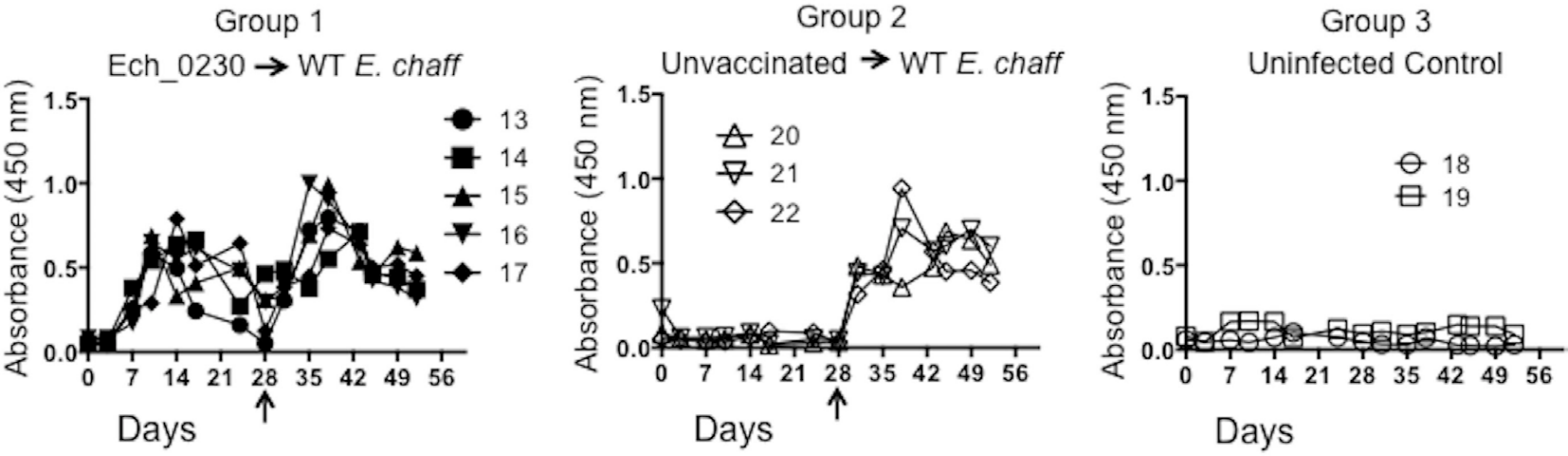
*Ehrlichia chaffeensis-*specific IgG response following Ech_0230 vaccination and secondary challenge with wild-type *E. chaffeensis*. Total *E. chaffeensis*-specific IgG was measured in the serum at multiple time points by ELISA in dogs vaccinated with the Ech_0230 mutant and challenged with wild-type *E. chaffeensis* (group 1), in unvaccinated control dogs that were infected with wild-type *E. chaffeensis* (group 2), and in uninfected negative control dogs (group 3). Dogs in groups 1 and 2 were challenged with virulent *E. chaffeensis* on day 28 post infection (indicated by the arrow). Each line is representative of a single animal. The data are not significantly different between groups as determined using two-way ANOVA with Sidak’s multiple comparisons test.

In our previous studies, vaccine-induced protection from wild-type *E. chaffeensis* infection correlated with the development of both humoral and cellular immune responses (9, 10). Therefore, we assessed the immune response in the Ech_0230 vaccinated dogs compared to unvaccinated control animals. *E. chaffeensis-*specific IgG concentrations were measured in the serum by indirect ELISAs. As seen in Figure 1, all five Ech_0230-vaccinated animals developed an *E. chaffeensis-*specific IgG response by 7–14 days after vaccination, and then antibody levels returned to low or baseline levels by day 28 after vaccination. Following wild-type challenge on day 28 after vaccination, the Ech_0230-vaccinated animals demonstrated a second peak in *E. chaffeensis*-specific IgG in the serum; however, this response was not significantly increased over the primary immune response, nor was it significantly increased compared to the IgG response observed in the unvaccinated control dogs (Figure 1).

**Figure 2:**
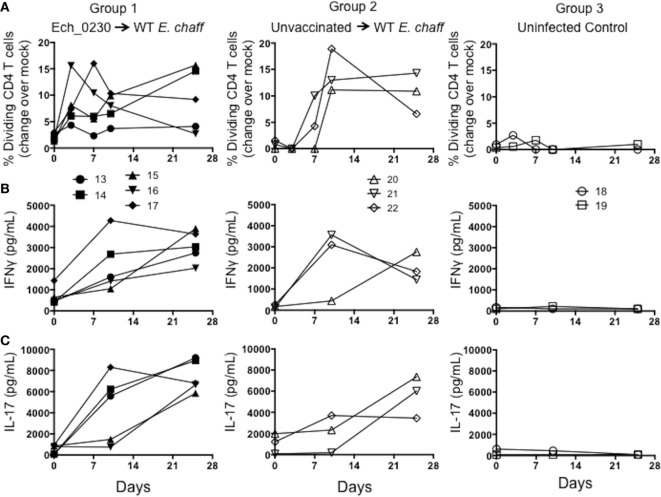
Cellular immune responses in dogs vaccinated with the Ech_0230 mutant and challenged with wild-type *Ehrlichia chaffeensis*. Peripheral blood mononuclear cell from dogs vaccinated with the Ech_0230 mutant and challenged with wild-type *E. chaffeensis* (group 1), unvaccinated control dogs that were infected with wild-type *E. chaffeensis* (group 2), and uninfected negative control dogs (group 3) were labeled with Cell Trace Violet, then cultured for 5 days at 4 × 10^5^ cells/well in the presence or absence of 10 μg/mL *E. chaffeensis* host cell-free lysate grown in the tick ISE6 cell line. On day 5, single-positive CD4^+^ T cells were analyzed by flow cytometry for Cell Trace Violet dilution as a measure of proliferation. **(A)** The percentage of CD4^+^ T cells that have proliferated in response to *E. chaffeensis* antigens as measured over the course of the experiment. The background (mock stimulated) proliferation was subtracted, and results represent change over mock. **(B,C)** Cell culture supernatants from the stimulated cell cultures were analyzed by commercial ELISA kit for **(B)** IFNγ and **(C)** IL-17. Each line is representative of a single animal. **(A–C)** The data are not significantly different between groups as determined using two-way ANOVA with Sidak’s multiple comparisons test.

We used *in vitro* antigen-recall assays to measure the *E. chaffeensis-*specific CD4 and CD8 T cell response in dogs vaccinated with the Ech_0230 mutant, and unvaccinated control animals. For proliferation assays, PBMCs were labeled with CellTrace violet and stimulated with host cell-free *E. chaffeensis* lysate for 5 days, and then CD4 and CD8 T cell proliferation was measured by flow cytometry. Representative gating strategies from a control unvaccinated animal and an uninfected control animal are depicted in Figure S1 in Supplementary Material. We were unable to detect *E. chaffeensis*-specific CD4 or CD8 T cell responses in Ech_0230-mutant vaccinated dogs prior to challenge (data not shown). Following wild-type challenge, both Ech_0230-vaccinated animals and control, unvaccinated animals mounted a significant cellular response. As seen in Figure 2A, dogs in both vaccinated and unvaccinated groups mounted a significant *E. chaffeensis*-specific CD4 T cell response by 7–10 days after infection. Consistent with our previous studies (10), we could not measure a significant, antigen-specific proliferative response by CD8 T cells from either group (data not shown). We also assessed antigen-specific cytokine secretion by PBMC by performing ELISAs on the 5-day stimulated cell culture supernatants. As seen in Figures 2B,C PBMC from animals in groups 2 and 3 secreted both IFNγ and IL-17 in specific response to host cell-free *E. chaffeensis* lysate on day 10 post challenge. The cellular immune response was sustained in both groups of animals, as we still observed significant proliferation and cytokine secretion even on day 25 post-infection. This sustained immune response is similar to what we have observed in previous studies (10), and is likely a result of persistent *E. chaffeensis* infection in these animals. In our prior studies, we have detected *E. chaffeensis* in the blood and tissues for as long as 42 days after infection (7). Neither CD4 T cell proliferation nor PBMC cytokine secretion differed significantly between Ech_0230-vaccinated animals and unvaccinated control animals, suggesting that the Ech_0230 vaccination did not induce a sufficiently robust cellular immune response in these animals to promote protection from virulent challenge.

Together, our results suggest that although the Ech_0230 mutant organism induced a transient increase in *E. chaffeensis-*specific IgG, it did not promote the development of a long-lived *E. chaffeensis*-specific memory response, nor did it confer protection from secondary, wild-type challenge with a virulent strain of *E. chaffeensis*.

### Canine CD4^+^CD8^+^ DP T Cells Expand in Response to Wild-Type *E. chaffeensis* Infection

**Figure 3:**
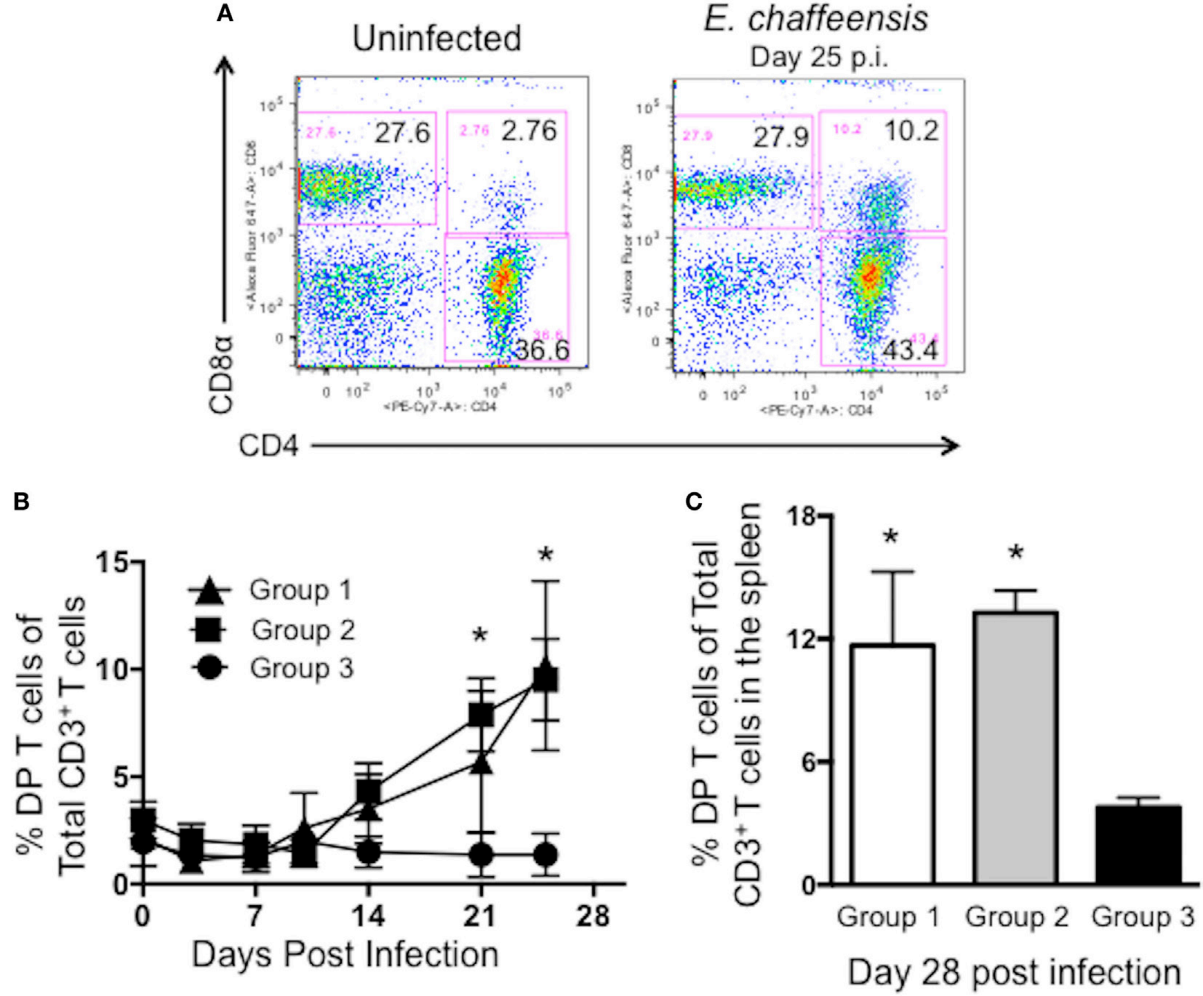
Canine CD4^+^CD8^+^ double-positive (DP) T cells expand in the peripheral blood and spleen during *Ehrlichia chaffeensis* infection. Peripheral blood was collected from dogs in groups 1–3 on days 0, 3, 7, 10, 14, 21, and 25 post-infection and analyzed by flow cytometry for the frequency of circulating CD3^+^CD4^+^CD8^+^ DP T cells. **(A)** Representative flow plots from an uninfected and *E. chaffeensis* infected dog on day 25 post-infection. Plots are gated on live cells, lymphocytes, and CD3^+^ cells. A representative gating strategy is depicted in Figure S1 in Supplementary Material. **(B)** Aggregate results from each dog collected over the course of the experiment. Group 1 was vaccinated with the Ech_0230 mutant and challenged with wild-type *E. chaffeensis*; group 2 was unvaccinated and infected with wild-type *E. chaffeensis*; and group 3 was uninfected. *p < 0.05 compared to uninfected controls (group 3) as determined using two-way ANOVA with Sidak’s multiple comparisons test. **(C)** Animals were euthanized on day 28 post-infection. Samples of spleen were processed into single cell suspensions and analyzed by flow cytometry for the frequency of live, CD3^+^CD4^+^CD8^+^ DP T cells among all CD3^+^ T cells. Data represent mean ± SEM. *p < 0.05 compared to uninfected controls (group 3) as determined by one-way ANOVA with Tukey’s post-test.

Dogs infected with wild-type *E. chaffeensis* develop a persistent infection. While it is clear that induction of cellular and humoral immunity has the capacity to prevent an infection in the dog model (9, 10), little is known about the immune response required for controlling an established infection in the natural host. Interestingly, in this study, we noted a significant expansion of DP T cells in the peripheral blood of control, wild-type *E. chaffeensis-*infected animals (group 2, Figure 3) and Ech_0230-mutant vaccinated dogs (group 1, Figure 3). Figure 3A depicts representative flow cytometry plots from a single uninfected control animal (group 3) and a wild-type *E. chaffeensis* infected animal (group 2). Figure 3B depicts the frequency of DP T cells in the blood of animals in groups 1–3 over the course of the infection. DP T cells comprise only 2–3% of all the T cells in normal animals; however, dogs that were persistently infected with wild-type *E. chaffeensis* developed frequencies as high as 15% DP T cells of all circulating T cells (group 1: mean 10.17 ± 3.96% SEM; group 2: mean of 9.51 ± 1.77% SEM) by day 25 post-infection. When gating our flow cytometry data, we confirmed that the DP T cell population did not fall outside of a singlet gate, and thus these cells are not doublets or flow cytometry artifacts. In preliminary flow cytometry studies, we also confirmed that this population was T cell derived by using dump channels for autofluorescence, and using canine-specific antibodies to measure expression of CD21 (a B cell marker), CD5 (a T cell marker), CD45 (a marker expressed by all lymphocytes), and CD18 (and integrin most commonly found on myeloid cells). Consistent with being a T lymphocyte population, the CD4^+^CD8^+^ DP T cell population was positive for expression of CD45 and CD5, and negative for expression of CD21 and CD18 (data not shown). As seen in Figure 3C, the frequency of DP T cells was also significantly increased in the spleen of *E. chaffeensis*-infected animals (groups 1 and 2) compared to uninfected control dogs (group 3). Importantly, a similar increase in DP T cells in the blood and tissues was not apparent in our earlier studies in animals that were vaccinated with the Ech_0660-mutant strain and protected from persistent infection (10); therefore, we hypothesized that DP T cells in the *E. chaffeensis*-infected dogs may contribute to control of the established, persistent infection.

### Canine DP T Cells Proliferate and Secrete IFNγ and IL-17 in Specific Response to *E. chaffeensis* Antigen

Importantly, because we observed no difference in the frequency or kinetics of the DP T cell response between animals in groups 1 and 2 (Ech_0230 vaccinated and challenged vs. unvaccinated, *E. chaffeensis* infected; Figures 3B,C), we combined the data for both groups for the remainder of our analyses, allowing us to assess a larger population (*n* = 8 animals total).

**Figure 4:**
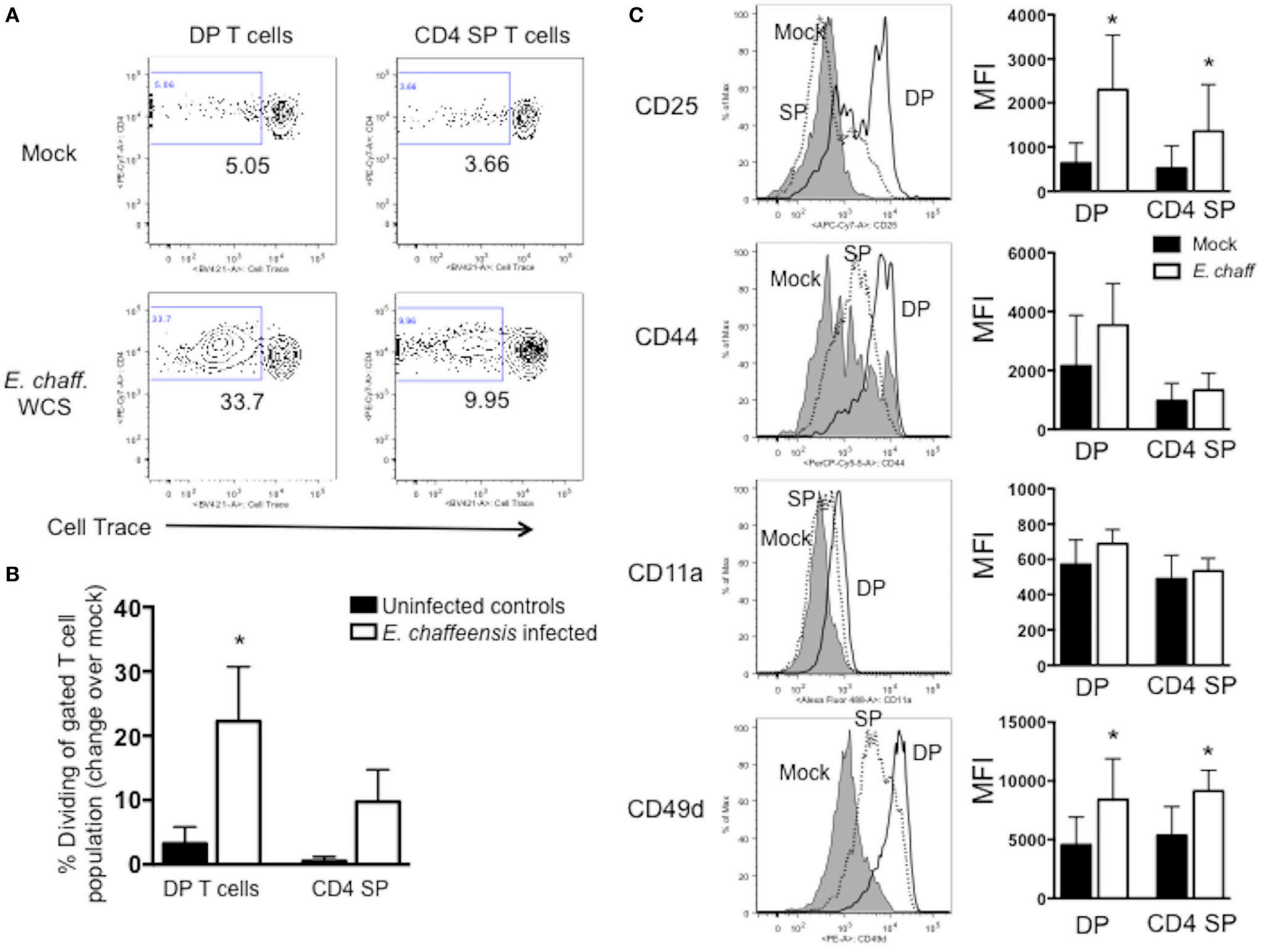
CD4^+^CD8^+^ double-positive (DP) T cells from *Ehrlichia chaffeensis* infected dogs proliferate and upregulate activation markers in specific response to *E. chaffeensis* antigen. On day 25 post-infection peripheral blood mononuclear cells were isolated from control, uninfected dogs and *E. chaffeensis* infected dogs in groups 1–3 as shown in Figure 2. Cells were labeled with Cell Trace Violet and stimulated for 5 days with 10 µg/ml *E. chaffeensis* lysate as in Figure 2. Mock-stimulated samples were used to correct for background proliferation. On day 5, antigen-specific CD3^+^CD4^+^CD8^+^ DP T cell and CD3^+^CD4^+^ SP T cell proliferation assessed by flow cytometry. Representative flow plots are shown in **(A)** and aggregate results are shown in **(B)**. Data represent mean ± SEM. *p < 0.05 compared to uninfected controls (group 3) as determined by one-way ANOVA with Tukey’s post-test. **(C)** After 5 days of stimulation with *E. chaffeensis* antigen as in **(A,B)**, CD3^+^CD4^+^CD8^+^ DP T cells and CD3^+^CD4^+^ single-positive (SP) T cells were analyzed for surface expression of CD25, CD44, CD11a, and CD49d. The left panel depicts representative flow plots from a single dog infected with *E. chaffeensis*, gated on live, CD3^+^CD4^+^CD8^+^ DP T cells. Gray histograms depict mock-stimulated DP T cells. Dotted histograms represent *E. chaffeensis*-stimulated CD4 SP T cells. Open histograms represent *E. chaffeensis* stimulated cells. The right panel depicts the mean fluorescence intensity (MFI) for each marker. The graphs represent aggregate results from *n* = 8 and are shown as mean ± SEM. *p < 0.05 compared to mock-stimulated cells of the same type, as determined by one-way ANOVA with Tukey’s post-test.

To determine if the expanded DP T cells we observed in our animals were antigen-specific, we next examined their capacity to proliferate in specific response to *E. chaffeensis*. PBMCs were isolated from the peripheral blood on day 21-post-infection, labeled with CellTrace Violet proliferation dye and stimulated for 5 days with host cell-free *E. chaffeensis* antigen as in Figure 2. On day 5, flow cytometry was used to measure DP and CD4 single-positive (SP) T cell proliferation. As seen in Figures 4A,B, a significant frequency of DP T cells from *E. chaffeensis* infected dogs underwent division in response to *E. chaffeensis* antigen, while DP T cells from control dogs did not. The expansion of DP T cells was similar to that observed for SP T cells from *E. chaffeensis*-infected dogs (Figure 4B). We did not observe a significant population of DP T cells undergoing division in control, unstimulated cultures (Figure 4A). DP T cells from both uninfected and *E. chaffeensis*-infected dogs underwent significant cell division in response to our positive control, ConA, and we observed no significant differences between the groups in their response to the mitogen (data not shown). Consistent with the observed proliferative response, DP T cells from infected dogs also upregulated surface expression of a number of T cell activation markers in response to *E. chaffeensis* antigen, including CD25, CD44, CD11a, and CD49d (Figure 4C).

**Figure 5:**
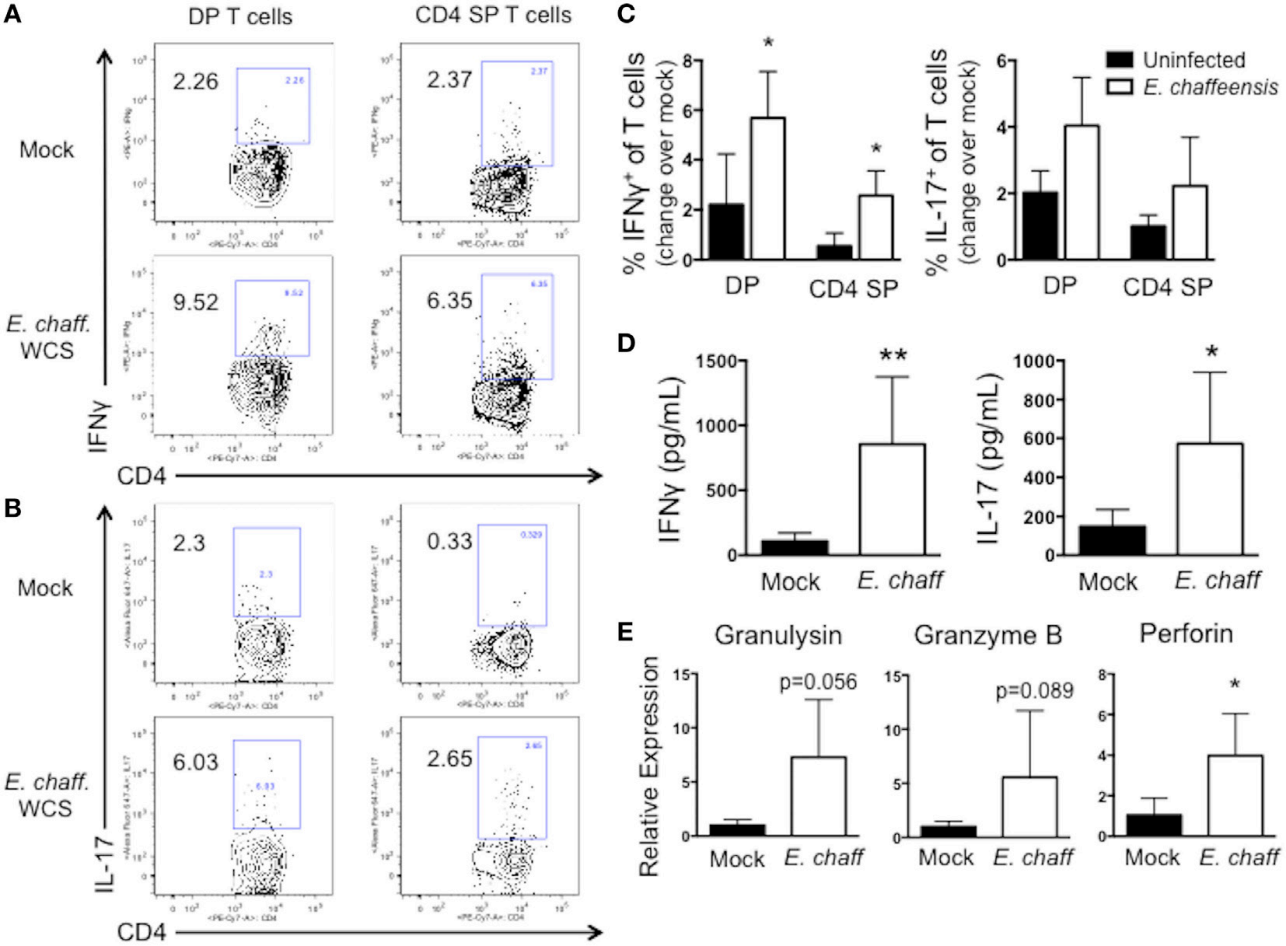
CD4^+^CD8^+^ double-positive (DP) T cells from *Ehrlichia chaffeensis* infected dogs secrete IFNγ and IL-17, and upregulate expression of granzyme B and granulysin, in response to *E. chaffeensis* antigen. **(A)** On day 25 post infection, peripheral blood mononuclear cells (PBMCs) were stimulated overnight with 10 µg/ml host cell-free *E. chaffeensis* lysate in the presence of Brefeldin A. Mock-stimulated samples were used to correct for background cytokine secretion. Cells were fixed and permeabilized, and the frequency of antigen-specific CD3^+^CD4^+^CD8^+^ DP T cells and CD3^+^CD4^+^ single-positive (SP) T cells producing IFNγ and IL-17 were assessed by intracellular cytokine staining. Representative flow plots for IFNγ staining are shown in **(A)** and for IL-17 are shown **(B)**. Aggregate results are depicted in **(C)**. *p < 0.05 compared to uninfected controls (group 3) as determined by one-way ANOVA with Tukey’s post-test. **(D)** On day 28 post-infection, CD3^+^CD4^+^CD8^+^ T cells were FACS purified from the peripheral blood of six *E. chaffeensis*-infected dogs and were cultured for 5 days with autologous monocytes in the presence or absence of 10 µg/ml *E. chaffeensis* antigen. Cell culture supernatants were then analyzed by commercial sandwich ELISA for IFNγ and IL-17. **p < 0.01, *p < 0.05 compared to mock-stimulated cells as determined by Student’s *t*-test. **(E)** On day 21 post infection, CD3^+^CD4^+^CD8^+^ T cells were FACS purified from the peripheral blood of six *E. chaffeensis* infected dogs and were cultured for 18 h with autologous monocytes and 10 µg/ml *E. chaffeensis* antigen. The RNA was isolated from the co-cultures and analyzed by qPCR for expression of granzyme B, granulysin, and perforin. Results were normalized to the housekeeping gene GAPDH and then expressed relative to unstimulated control cultures. Data represent mean ± SEM. *p < 0.05 compared to mock-stimulated cells as determined by Student’s *t*-test.

We next examined the ability of DP T cells to secrete cytokines in response to *E. chaffeensis* infection. PBMCs were stimulated with *E. chaffeensis* antigen in the presence of brefeldin A, and then intracellular cytokine staining was performed for IFNγ and IL-17. PBMC from *E. chaffeensis*-infected dogs demonstrated an increased frequency of IFNγ^+^ and IL-17^+^ DP and SP T cells compared to uninfected controls (Figures 5A–C), although the increase in IFNγ-producing cells was not statistically significant.

We confirmed the results of our intracellular cytokine staining using ELISAs. DP T cells were sort-purified from *E. chaffeensis* infected dogs, and cultured for 5 days with *E. chaffeensis* antigen in the presence of autologous monocytes. On day 5, stimulated cell supernatants were collected and analyzed by ELISA for IFNγ and IL-17. As seen in Figure 5D DP T cells secreted significant concentrations of both IL-17 and IFNγ in specific response to *E. chaffeensis* antigen.

Double-positive T cells from humans participate in cytotoxic responses and have the capacity to eliminate HIV-infected target cells (31), as well as cancer cells (32). Therefore, to determine the cytotoxic potential of DP T cells in the canine host, we used a qPCR assay to measure expression of the cytotoxic molecules granulysin, granzyme B, and perforin by the purified DP T cell:monocyte co-cultures responding to *E. chaffeensis* infection (Figure 5E). We observed an increase in expression of all three transcripts in response to *E. chaffeensis* antigen, although the increase was only statistically significant for perforin.

## Discussion

We have previously reported the generation of both random and targeted mutations in *E. chaffeensis*, and established three stable transposon insertion mutants that demonstrated a deficiency in their ability to infect vertebrate hosts (8, 13). Infection with one of these mutants, the Ech_0660 mutant clone, was able to confer protection from wild-type *E. chaffeensis* infection (9, 10). Given our success using the attenuated Ech_0660 mutant organism as a vaccine, we examined the ability of the Ech_0230 mutant to confer protection against secondary wild-type *E. chaffeensis* challenge. Surprisingly, Ech_0230 vaccination did not protect the animals from subsequent wild-type challenge. It is currently unclear why the Ech_0230 mutant failed to protect; however, we did observe notable differences in the host immune response induced by Ech_0660 vaccination versus that induced in the Ech_0230-vaccinated dogs. Vaccination with the Ech_0660 mutant induces an IgG response in the serum (9, 10), and a CD4 T cell response that is detectable in the peripheral blood by 7–14 days after infection (10). In contrast, the Ech_0230 mutant induced a transient IgG response in the serum, but did not elicit cellular immune responses in the blood. Following challenge, the Ech_0660 animals demonstrated a rapid, amnestic humoral, and cellular response following wild-type challenge (9, 10), while the response in the Ech_0230-vaccinated animals did not. The duration of antigen exposure and the inflammatory environment are both key variables influencing the generation of immune memory (45, 46). In support of this, there are many reports demonstrating that rapid pathogen clearance, either through actions of the innate immune system or through antibiotic therapy, results in a truncated primary immune response and failure to establish long-lived sterilizing immunity (46–49). Although the Ech_0660 mutant is rapidly cleared, it can be detected in the peripheral blood in some animals for as long as 5 days (9, 10). In contrast, the Ech_0230 mutant is undetectable even 1 day after inoculation [Table 1; (8, 13)]. Thus, it is feasible that the slightly sustained presence of the Ech_0660 organism is more effective at inducing long-lived immune memory.

This report is, to our knowledge, the first description of a DP T cell population responding to *Ehrlichia* infection. A number of studies have analyzed the T cell response to *E. chaffeensis* and related *Ehrlichia* species using murine models (16–23, 25, 50–52); and although mice do possess DP T cells (26), we have found no reports of this population contributing to the immune response to rickettsial infection. However, there have been previous reports of other nonconventional lymphocyte populations responding to *Ehrlichia* infection, such as NKT cells (24, 25), and as reported by our own group, CD4*^−^*CD8^−^ double negative T helper cells (20). It is unlikely that the cells we have identified in our studies are NKT cells, as the population has not been described to express both CD4 and CD8; and in our own previous report, CD4*^−^*CD8*^−^* double negative T helper cells were increased in CD4 T cell-deficient mice (20), which are known to carry an expanded CD4*^−^*CD8*^−^* helper T cell population (53).

Double-positive T cells in humans are most commonly associated with chronic disease conditions, such as hepatitis C virus infection (54), HIV infection (31), and cancer (32, 33). Thus, the persistence of antigen may be a prerequisite for the development of this unique population. In dogs, less is known about DP T cells during disease states; however, Alexandre-Pires et al. observed an increase in DP T cells in dogs with leishmaniasis, a chronic parasitic disease (35). *E. chaffeensis* infection causes a persistent infection, with ehrlichemia detectable for at least 42 days after infection (7); thus it may not be surprising that we observed a significant expansion of DP T cells in our animals. One caveat to our studies is the high infection dose and route of inoculation. We chose to challenge our dogs with a high intravenous dose of *E. chaffeensis*, because this stringent challenge model has been successful in our prior studies for determining the efficacy of our experimental live-attenuated vaccines (9–11). However, intravenous inoculation is not a physiologic infection route, and it is also unlikely that an *E. chaffeensis* infected tick would transmit such a high dose or organisms, although the range of organisms transmitted from an infected tick remains to be established. Therefore, DP T cells may arise in our animals due to the significant stress on their immune system. Importantly, however, we did not observe such a population develop in our studies using the attenuated Ech_0660 vaccine (10), in which the dogs were protected from infection, despite using intravenous inoculation and a high challenge dose.

At this time, it is unclear if DP T cells are beneficial or detrimental to the *E. chaffeensis*-infected host. As stated above, dogs in our previous studies that were vaccinated with the Ech_0660 mutant and subsequently protected from wild-type *E. chaffeensis* challenge (10), did not demonstrate a significant increase in the prevalence of DP T cells in the blood or organs. Further, in animals that clear the infection, such as mice, we have found no reports of the development of antigen-specific DP T cells. Thus, it is unlikely that DP T cells are critical for resistance to *E. chaffeensis* infection. However, given their capacity for cytokine production and cytotoxicity, it is possible that DP T cells contribute to disease control following establishment of a persistent *E. chaffeensis* infection, which occurs in the canine host while absent in the murine host. Alternatively, however, DP T cells may instead contribute to disease pathology, or are indicative of an early stage of immune exhaustion. A series of recent papers have suggested that human patients with chronic *Trypanasoma cruzi* infection develop a population of CD4^+^CD8^+^ DP T cells, and that their appearance correlates with immune exhaustion (55, 56). Similar findings have also been reported during HIV infection (57). It is currently unknown if persistent *E. chaffeensis* infection leads to immune exhaustion in the dog. The importance of DP T cells in immunity to persistent *E. chaffeensis* infection will be the subject of future studies in our laboratory.

In summary, we have reported, for the first time, the significant expansion of a pathogen-specific DP T cell population in dogs that are persistently infected with *E. chaffeensis*. This population has the capacity for cytokine secretion and cytotoxicity, and thus has potential to contribute to disease outcome during tick-borne infection. In addition to vector-borne diseases caused by pathogens in the genera *Ehrlichia, Anaplasma*, and *Rickettsia*, dogs are susceptible to many of the same chronic disease conditions as humans, including rheumatoid arthritis, *Leishmania* infection and several cancers. Given the similarities described between human and canine DP T cells, the dog may represent an excellent model to further elucidate the role of this rare population in disease pathogenesis and immunity.

## Ethics Statement

Experimental procedures were performed in strict compliance with federal and institutional guidelines and were approved by the Kansas State University Institutional Animal Care and Use Committee.

## Author Contributions

JM, YW, and RG designed the experiments. JM, YW, GB, CG, and RG performed the experiments and analyzed the data. JM, YW, and RG wrote the manuscript. All authors reviewed the manuscript.

## Conflict of Interest Statement

The authors declare that the research was conducted in the absence of any commercial or financial relationships that could be construed as a potential conflict of interest.
