# Supplementary material for: Antigen-Specific CD4+CD8+ Double-Positive T Cells Are Increased in the Blood and Spleen During Ehrlichia chaffeensis Infection in the Canine Host
Source: Front Immunol. 2018 Jul 11;9:1585. doi: 10.3389/fimmu.2018.01585 (PMC6050357; doi:10.3389/fimmu.2018.01585)
Supplement: Supplementary file 1 [file image_1.PDF]

Supplementary Material

Antigen-specific $CD4^+CD8^+$ Double Positive T cells are increased in the blood and spleen during *Ehrlichia chaffeensis* infection in the canine host

Jodi L. McGill[1#], Ying Wang[2#], Chanran K. Ganta[2], Gunavanthi D. Y. Boorgula[2], Roman R. Ganta[2]

[1]Department of Veterinary Microbiology and Preventative Medicine, College of Veterinary Medicine, Iowa State University, Ames, Iowa, USA

[2]Center of Excellence for Vector-Borne Diseases, Department of Diagnostic Medicine/Pathobiology, College of Veterinary Medicine, Kansas State University, Manhattan, Kansas, USA

Correspondence: Corresponding Author: jlmcgill@iastate.edu

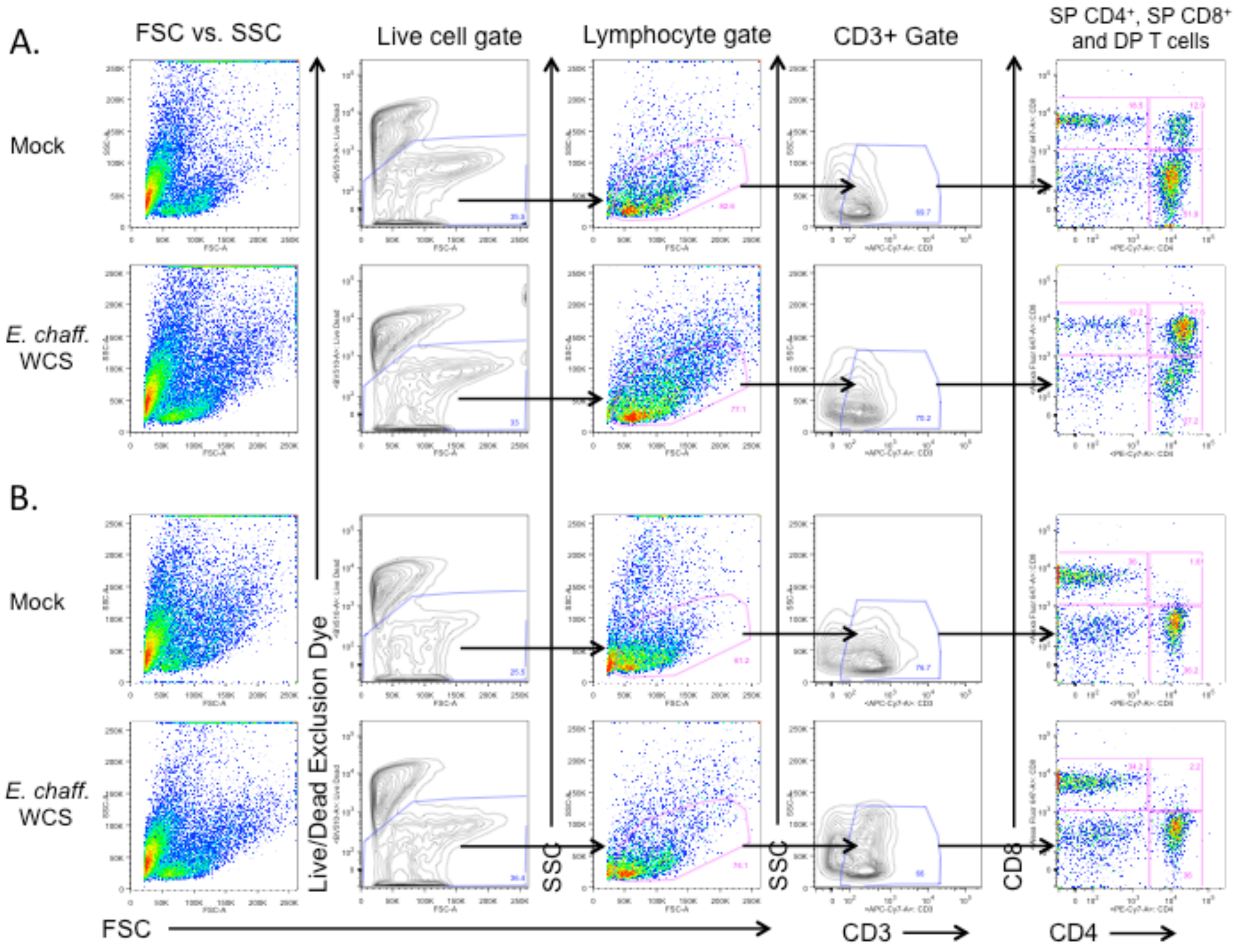

Supplementary Figure 1. Gating Strategy for CD4$^+$ single positive T cells and CD4+CD8+ Double Positive T cells. Representative gating strategies are shown from (A) an *E. chaffeensis* infected (group 2) and (B) an uninfected dog (group 3) on day 25 post infection. PBMC remained unstimulated (mock) or were stimulated with whole cell sonicate from *E. chaffeensis* as described in Materials and Methods. Flow plots were gated on live cells, lymphocytes and CD3$^+$ cells, and then CD4 single positive, CD8 single positive or CD4+CD8+ double positive T cells were gated for further analysis.
